# Sea cucumber polypeptide ameliorates aging properties via the brain-gut axis in naturally aging mice

**DOI:** 10.1186/s13020-025-01201-2

**Published:** 2025-08-29

**Authors:** Chao Feng, Yiwen Shou, Shulin Wu, Han Mo, Xin Mao, Huisha Huang, Qinpei Lu, Li Xia, Lu Lu, Zhiheng Su, Hongwei Guo, Zhaoquan Huang

**Affiliations:** 1 Department of Pathology, The First Affiliated Hospital of Guangxi Medical University, Nanning, 530021 Guangxi China; 2 Guangxi Key Laboratory for Bioactive Molecules Research and Evaluation & College of Pharmacy, Guangxi Medical University, Nanning, 530021 Guangxi China; 3 Key Laboratory of Longevity and Aging-Related Diseases of Chinese Ministry of Education & Center for Translational Medicine, Guangxi Medical University, Nanning, 530021 Guangxi China; 4 Center for Genomic and Personalized Medicine, Guangxi Medical University, Nanning, 530021 Guangxi China

**Keywords:** Sea cucumber polypeptide, Aging, Brain-Gut axis, Metabonomic, Naturally aging mice

## Abstract

**Background:**

Sea cucumber has been recognized as a traditional nutraceutical in Chinese medicine for millennia, with its derived polypeptide (SCP) demonstrating diverse bioactive properties. Nevertheless, the molecular mechanisms underlying SCP's potential geroprotective effects remain insufficiently characterized.

**Methods:**

We systematically evaluated SCP's impact on neuromotor function and cognitive performance in physiologically aged C57BL/6 J mice models using a behavioral test battery comprising open field, Y-maze, and Barnes maze paradigms. Complementary multi-omics approaches were employed to interrogate age-related perturbations in gut microbial ecology (16S rRNA sequencing) and systemic metabolism (untargeted LC–MS).H&E and immumohistochemical staining was used to evaluate the pathological features of mice brain tissues and intestinal tissue. Bulk RNA-sequencing was used to detect gene expression profiles in mice brain tissue.

**Results:**

Behavioral assessments (open field, Y-maze, Barnes maze) demonstrated that SCP intervention effectively delayed the decline in exercise, learning and memory abilities in aging mice. SCP administration enhanced cerebral organosomatic indices and hepatic functional markers while reducing neuronal senescence biomarkers. Furthermore, SCP improved intestinal mucosal barrier function in aging mice restored gut microbial diversity metrics, effectively counteracting age-associated dysbiosis. Mechanistically, SCP induced taxonomic restructuring characterized by increased abundance of neuroprotective Eubacterium_brachy_group and Prevotellaceae genera, concomitant with suppression of dementia-linked Dubosiella. Metabolomic integration revealed SCP-mediated upregulation of steroidogenic pathways correlating with cognitive enhancement. Multi-omics validation through integrated transcriptomic profiling and immunohistochemical quantification corroborated these physiological improvements.

**Conclusion:**

Our findings propose a mechanism whereby SCP might exert geroprotective effects through multimodal regulation of the gut-brain axis and systemic metabolic homeostasis, establishing mechanistic foundations for its translational potential in healthy longevity promotion.

**Supplementary Information:**

The online version contains supplementary material available at 10.1186/s13020-025-01201-2.

## Background

In recent years, global demographic analyses have witnessed a progressive aging of populations, a phenomenon evident not only in industrialized nations but also on the rise in developing regions. As of the conclusion of 2022, China had approximately 280 million geriatric population (aged ≥ 60 years), accounting for 19.8% of the total demographic composition. This accelerating demographic aging trend imposes a substantial societal burden [1]. Aging constitutes an inevitable and irreversible degenerative process in organisms, characterized by physical changes and a decline in organ function [2]. Consequently, the development of anti-aging therapeutics has garnered considerable attention, emphasizing the importance of identifying safe interventions capable of modulating aging processes, particularly those exhibiting both pharmacotherapeutic and nutraceutical properties.

Sea cucumber, an echinoderm in the Holothuroidea class, predominantly distributes in the intertidal and shallow benthic ecosystems [3]. Traditionally, sea cucumber has been historically documented as a tonifying agent in Chinese medical literature. Its bioactive components encompass polypeptides, polysaccharides, lipids, triterpenoid saponins, lectin, neuropeptides, and glycopeptides [4]. The body wall of sea cucumber demonstrates excellent protein richness, with concentrations reaching approximately 70% of dry weight, providing a complete profile of essential amino acids that fulfill human nutritional requirements, thereby constituting an excellent source of high-quality, bioavailable protein [5]. Additionally, sea cucumber exhibits a rich profile of essential trace elements, including iron, copper, molybdenum, and selenium. Studies have highlighted the diverse biological activities of sea cucumber polypeptides (SCPs), including antioxidative, antihypertensive, neuroprotective, alleviating gut microbiota imbalance, antiaging, wound-healing, anti-hyperuricemia, antitumor, and immunomodulatory effects [6–9]. Due to their safety profile, ease of absorption, potent biological activity, and plentiful resources, SCPs demonstrate significant potential for nutraceutical development and therapeutic applications.

In the present study, we aimed to investigate the efficacy of SCP in delaying aging and delineate their underlying molecular mechanisms. Utilizing naturally aging C57BL/6 J mice as a model, we pharmacologically evaluated the enhancement of motor function, memory, and learning capabilities following SCP consumption. Furthermore, we conducted analyses on gut microbiota and metabolomics to assess alterations in the gut ecosystem and metabolic profiles of aging mice. The experimental results of this research shed light on the anti-aging properties of SCP, substantiating its translational potential in developing functional foods and medications aimed at slowing down the aging process.

## Materials and methods

### Identification of peptides in SCP and its molecular weight distribution and amino acids profile.

**Fig. 1:**
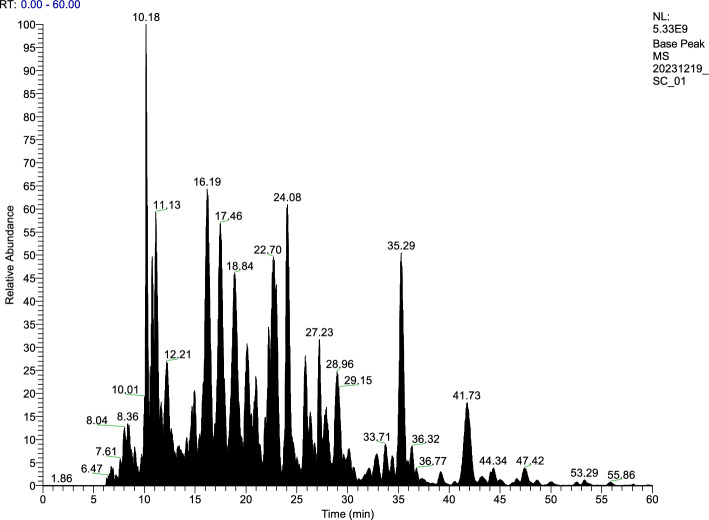
Mass spectrum of each component in SCP

SCP was pharmacologically grade and supplied by Dalian Shenlan Peptide Technology Research and Development Co., LTD (Dalian, China). Peptide sequences of SCP were characterized by liquid chromatography mass spectrometry (LC–MS) and subsequent bioinformatic alignment with the sea cucumber proteomic database was performed to identify predominant peptide constituents. A total of 308 protein groups were identified (Fig. 1 and Table 1). Using high-performance size exclusion chromatography, the molecular weight distribution profile of SCP was characterized. The chromatographic analysis was performed on a Waters 600 high performance liquid chromatography (HPLC) system equipped with a tosoh silica (TSK) gel column (2000 SWXL, 300 mm × 7.8 mm), and a 2487 ultraviolet (UV). The elution was carried out at 30℃ using a mobile phase consisting of water/acetonitrile/trifluoroacetic acid (85:15:0.05, v/v/v) delivered at a flow rate of 0.7 mL/min. Cytochrome c (12,500 Da), aprotinin (6512 Da), β-amyloid (4514 Da), vitamin B12 (1355 Da), MOG (2582 Da), GSH (307 Da), and glycine were among the standards used for calibration. The molecular weight calibration curve was established by plotting the relationship of logarithmic molecular weight(y) versus retention time(x), yielding the equation y = 0.0162x + 0.0197 with a correlation coefficient(R^2^) of 0.9992, demonstrating excellent linearity across the analytical range. The relative molecular weight distribution profile of the peptide constituents was subsequently determined by applying this equation, with quantitative analysis of each fraction achieved through normalized peak area integration (Table S1). Additionally, an automatic amino acid analyzer (Model 835–50, Hitachi, Tokyo, Japan) was quantitatively used to analyze the amino acid composition of the SCP (Table S2).

**Table 1:** The main peptide sequence in SCP

Accession	Peptide sequence
A0A9Q1BZ59	GPAGPQGPQGPAG
	GPAGPTGPTGPA
	GPAGPQGPQ
	GPAGPQGPAG
	DQGAPGAPGEQGE
A0A1I9W676	GPAGPTGPTGPA
	GPAGPQGPAG
	GPAGPSGPPGP
	RGPAGPTGPTGPA
	PRGPPGAP
A0A9Q1H7K3	KIVPGAPDDV
	GRDGETGPGGATGPAG
	QGPPGPPGEP
	GPAGLNGDRGAPG
	GEPGAQGNRGPDGPAGAPG
A0A9Q1H0A2	VGSDGVPGRE
	VGPNDPDHAN
	MDYDERPNN
	VGPNDPDHA
A0A368NM68	GSNMLFHNGAGEQ
	GSNMLFHNGAGE
A0A2G8K2G2	FDQADDPRPT
	DQADDPRPT
A0A9Q1C1P7	SQPAGGQPPQ
	QPAGGQPPQ
	PGQPPSQPAGGQ
A0A1E3X206	APGAPGGAVMSALG
	APGGAVMSALG
	GAPGGAVMSALG

### Experimental animal design

Following a one-week acclimation period under controlled vivarium conditions(24 ± 1 °C, 60 ± 5% relative humidity, 12:12 h light–dark cycle), six 8-week-old C57Bl/6 J mice and eighteen 20-month-old C57Bl/6 J mice were prepared for subsequent interventions. Throughout the acclimatization period, animals were provided unrestricted access to food and water. After 7 days of environmental adaptation, the mice were randomly allocated into four experimental groups(n = 6/group):

- (i) The Young-control Group(8 weeks): Received a daily oral dose of 0.2 mL of saline.
- (ii) The Aging-Control Group(20 months): Received a daily oral dose of 0.2 mL of saline.
- (iii) The Aging-SCP Low Dose Group(20 months): Received a daily oral dose of 0.2 mL of SCP at a concentration of 500 mg/kg body weight.
- (iv) The Aging-SCP High Dose Group(20 months): Received a daily oral dose of 0.2 mL of SCP at a concentration of 1000 mg/kg body weight.

Upon completion of behavioral assessments, the mice were humanely euthanized, and their brains were immediately excised, frozen in liquid nitrogen, and kept at −80℃ until subsequent biochemical analyses. C57Bl/6 J mice were procured from Beijing Weitong Lihua Experimental Animal Technology Co., Ltd.(Beijing, China).

### Weight and organ index assays

Throughout the experimental intervention period, mice body weights were measured biweekly, and any changes were meticulously documented. Post-euthanasia, the kidney, liver, heart, lungs, and brain of each mouse were precisely weighed to calculate their respective organ indexes, which were determined using the following formula: tissue index = (tissue weight/body weight) × 100%.

### Open field test

Each mouse was gently introduced into the center in an open field arena, consisting of a wooden box measuring 40 × 40 × 50 cm, allowing unrestricted exploration for a duration of 5 min. All behavioral sessions were digitally recorded using a camera. To quantitatively evaluate motor ability and anxiety, the total distance traveled, the distance covered within the center zone, and the number of entries into the center zone were calculated using the Smart Video Tracking Software(Panlab; Harvard Apparatus).

### Y maze

The Y-maze contraption consisted of three identical opaque polypropylene arms(35 × 20 × 10 cm; 120° angular separation) with matte white interior surfaces, constructed to minimize external visual cues and ensure standardized spatial navigation testing conditions. The floor was systematically decontaminated with 75%(v/v) ethanol solution to eliminate residual olfactory cues in between experiments. Throughout the test, consistent spatial visual cues were permanently affixed at the end of each maze arm. Through integration of distal spatial cues differentially perceived from each Y-maze arm, the mouse successfully discriminated between novelty and familiarity. The mice were permitted 3-min free exploration of two accessible maze arms, while the third arm remained obstructed by an opaque polycarbonate partition. Following a 120-min intertrial interval, the mice were reintroduced to the initial arm for the second experiment, with exploration behavior quantified during a 180-s epoch corresponding to each maze arm under unconstrained access conditions. For every mouse, the initial and novel arms were assigned randomly to control for positional bias across trials. When all four limbs entered the arm, the ingress was operationally defined as valid. Using Smart Video Tracking Software(Panlab; Harvard Apparatus), the animals'movements were tracked in order to assess their cognitive ability.

### Barnes maze

The Barnes maze apparatus was conducted on an elevated circular platform(100 cm diameter), featuring 20 equidistant holes(each 5 × 5 cm). One hole, randomly selected, served as the escape hole, allowing the mouse to descend into a dark box positioned beneath the platform. During the habituation phase(pre-test acclimation), the mouse was gently placed inside the dark box at the maze's center and allowed to remain there for 15 s. Following removal of the box, the mouse was permitted 180-s unrestricted exploration of the maze apparatus. During that time, the frequency of erroneous hole explorations and the escape latency were noted. The mouse was gently steered in the direction of the dark box if it failing to locate the target escape hole within the 180-s trial period. The mice underwent twice-daily training sessions for six consecutive days, and on the day 7, they were assessed. The maze apparatus was thoroughly cleaned with 75% ethanol between trials to eliminate residual olfactory cues. On day 7, the mice performed the task. The escape latency for the mouse to successful entry into the target box and the percentage of incorrect hole entries within the goal quadrant over a 3-min period were calculated. These metrics served to assess spatial reference learning and memory. All data were digitally acquired using a camera and analyzed objectively with an automated video tracking system(EthoVision 2.3 and XT 6.1.326, Noldus Information Technology, Wageningen, The Netherlands).

### Fecal metabolomics analysis

For fecal metabolomic profiling, the ultra-performance liquid chromatography coupled with quadrupole time of flight mass spectrometry(UPLC-QTOF-MS) technology was used based on its remarkable sensitivity, quick acquisition rates, and accuracy in mass data collection. Each 50 mg fecal sample was subjected to ultrasonic-assisted extraction to expedite the extraction procedure, followed by addition of 400 μl methanol containing 5 μg/ml of 2-chloro-L-phenylalanine as an internal standard for subsequently added. Following centrifugation(12,000 × g, 10 min, 4 °C), the resultant supernatant was moved to a glass container after centrifugation. An Agilent 1290 Infinity II ultra-high-performance liquid chromatography(UHPLC) system, an Agilent 6545 UHD, and an Accurate-Mass Q-TOF–MS(Agilent, Santa Clara, CA, USA) made up the liquid chromatography tandem mass spectrometry(LC–MS/MS) system. Raw MS data(.raw files) were processed using LECO Chroma TOF 4.3 × software(LECO Corporation),(then compared to the LECO-Fiehn Rtx5 database. Retention times and m/z data pairs were utilized to identify each peak, and only peaks having a matching probability of ≥ 60% were classified as confidently identified metabolites. The SIMCA14.1 software package was used to import the peaks, their numbers, sample names, and normalized peak areas. The Kyoto Encyclopedia of Genes and Genomes(KEGG) database was consulted in order to confirm the identified metabolites. All statistically significant metabolites were subjected to pathway topology analysis using MetaboAnalyst, with subsequent cross-referencing against canonical metabolic pathways in the KEGG database. To assess any associations between fecal metabolites and gut microbiota, Pearson's correlation coefficients was performed.

### Intestinal microbiota sequencing

Microbiome sequencing and analysis were outsourced to Shanghai Majorbio Biopharm Technology Co., Ltd(Shanghai, China). The first step was to use a Qiagen Gel Extraction Kit(Qiagen, Germany) to extract genomic (DNA from the intestinal contents as directed by the manufacturer. Following that, bar-coded primers that specifically targeted the V3-V4 region of the 16S rRNA gene were used to amplify DNA fragments by polymerase chain reaction(PCR). Following electrophoretic verification of the PCR results, the Thermo Scientific GeneJET Glue Recovery Kit was used to recover and purify the appropriate bands. Qualified libraries were subsequently subjected to sequencing on the NovaSeq 6000(Beijing, China).

### Real-time reverse transcription quantitative polymerase chain reaction(RT-qPCR) analysis

Total RNA was isolated from brain tissues using TRIZOL reagent(Takara, Shiga-ken, Japan) following the manufacturer's protocol., Subsequently, 1 μg of total RNA was reverse transcribed into cDNA for subsequent RT-qPCR analysis. Gene-specific primers were constructed applying Primer 6.0 software and synthesized by Sangon Biotech Co., Ltd.(Shanghai, China). RT-qPCR was performed in 20 μL reaction volumes using the following thermal cycling protocol: initial denaturation at 95 °C for 10 min; 45 cycles of denaturation at 95 °C for 10 s, annealing at 60 °C for 10 s, and extension at 72 °C for 10 s; with final fluorescence acquisition at 72 °C. In Stage III, melting curve analysis was performed with the following parameters: initial denaturation at 95 °C for 10 s, increasing gradually over 60 s to 65℃, followed by a final 1-s denaturation at 97 °C. The internal control for normalization was β-Actin. The 2^−ΔΔCt^ technique was used to examine the levels of messenger ribonucleic acid(mRNA) expression. The relevant primer sequences are listed in Table S3.

### Hematoxylin‐eosin(H&E) staining

The kidney, liver, heart, lungs, spleen and brain tissues harvested from each animal were immersion-fixed in a 4% paraformaldehyde solution for a duration of 24 h. Following fixation, the tissues were processed through graded ethanol dehydration(70%−100%), cleared in xylene, embedded in paraffin, and sectioned to a thickness of 5 μm(Servicebio Biological Technology Co., Ltd.). H&E staining was carried out utilizing an H&E staining kit sourced(Solarbio, China), in strict adherence to the manufacturer's protocols. The histopathological evaluation within the tissues were examined under a light microscope.

### Serum biochemical

Fresh whole blood samples were allowed to coagulate for 2 h at room temperature, followed by centrifugation at 3,000 × g for 15 min at 4 °C to obtain serum supernatant fractions. Following collection, all materials were kept at −80℃ for further scientific examination. The serum levels of alkaline phosphatase(ALP) and aspartate aminotransferase(AST) were quantitatively determined using standardized commercial assay kits. All biochemical analyses were performed strictly adhering to the manufacturer's recommendations on an automatic biochemical analyzer(Hitachi 7020, Japan).

### RNA sequencing

Brain tissue samples from groups(n = 3 per group) were randomly selected and frozen for subsequent RNA extraction, and then the samples were isolated for their purity, quality, and integrity. mRNA was enriched using oligo(dT)25 magnetic beads, and cDNA and libraries were prepared by Major Bio-Tech Ltd(Shanghai, China). RNA sequencing libraries were prepared following the Illumina protocols to get the reads, and then these reads were quantified with read numbers mapped to each gene using HTSeq v0.6.1. Pathway scores were assessed utilizing the Gene Set Variation Analysis(GSVA), followed by ordinal regression analysis. Pathway enrichment scores were computed using GSVA by integrating the normalized protein expression profiles of all constituent proteins within the especial KEGG-defined pathway.

### Alcian blue (AB) staining

Serial 5-μm brain sections were mounted on slides after being floated in a 40 °C water bath. Following oven drying at 60 °C for 2 h, tissue sections underwent sequential deparaffinization in xylene(3 × 5 min) and rehydration through graded ethanol series(100%−70%). Then, staining was carried out using the Arixin Blue staining kit.

### Immunohistochemical (IHC) staining

Serial 5-μm brain sections were mounted on slides after being floated in a 40 °C water bath. Following oven drying at 60 °C for 2 h, tissue sections underwent sequential deparaffinization in xylene(3 × 5 min) and rehydration through graded ethanol series(100%−70%). After that, sections were blocked with endogenous peroxidase, followed by antigen retrieval and nonspecific binding reduction. Tissue sections were incubated overnight at 4 °C with the following primary antibodies: anti COMT(1:100), anti UGT1A9(1:100), and anti HSD11B1(1:100). Following primary antibody incubation, the sections were incubated at room temperature with secondary antibody for 1 h and performed with staining agent. The expression of proteins was graded according to the staining intensity score(0, 1, 2 or 3) and positive rate score(0, 1, 2, 3 or 4). The composite score was calculated by multiplying the staining intensity score by the positive rate score.

### Statistical analyses

Student's t-test and one-way ANOVA analysis were performed to examine all results with SPSS 21 software(IBM Inc., Armonk, NY, USA). p < 0.05 was considered statistically significant. The association between fecal metabolomic profiles and gut microbial taxa were assessed using Pearson correlation analysis. All experimental data are presented as mean ± standard deviation(SD) and were visualized using GraphPad Prism 9.1.0(GraphPad Software, USA).

## Results

### SCP ameliorates age-associated decline in hepatic and neurological function

**Fig. 2:**
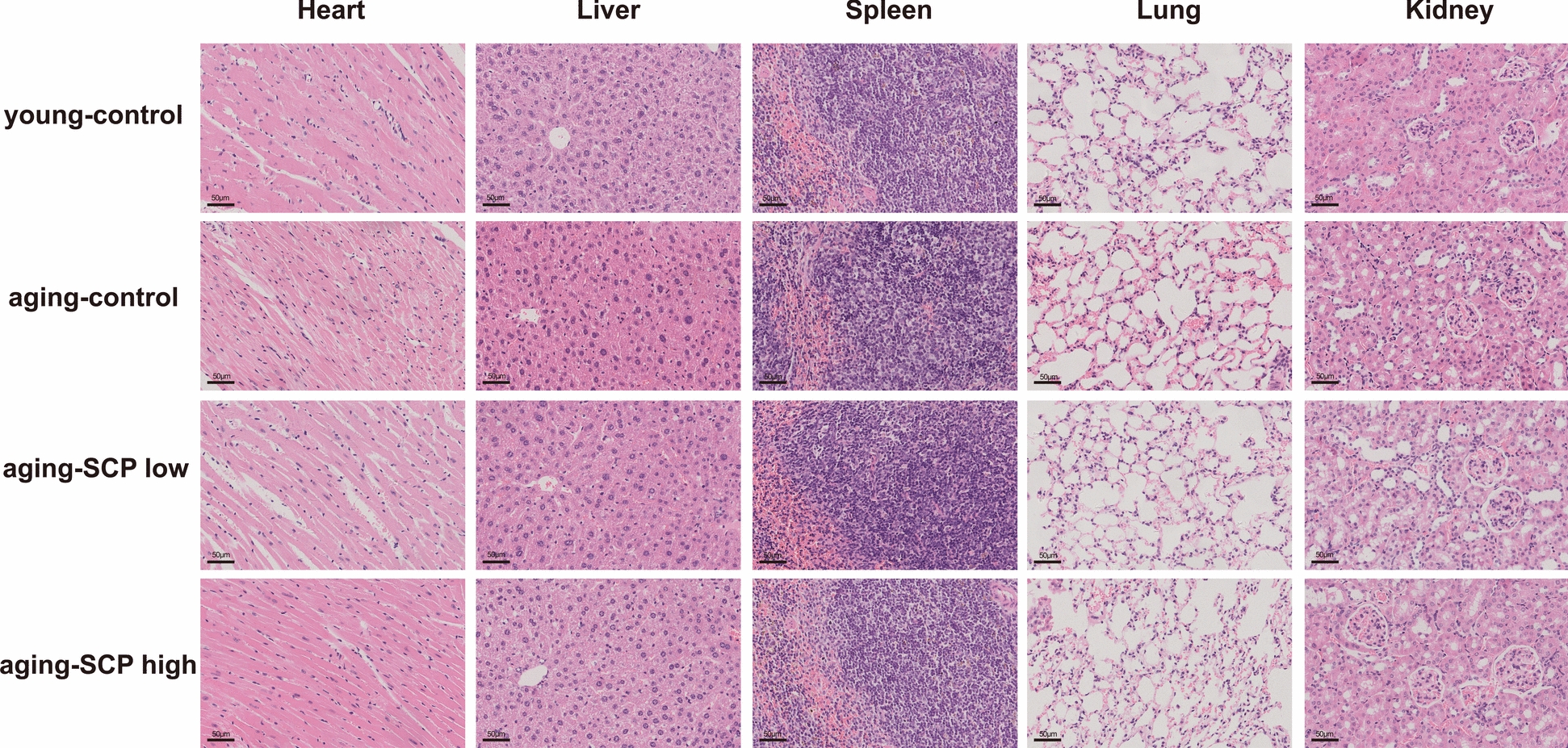
H&E staining images of heart, liver, spleen, lung and kidney of mice. Scale bar: 50 μm

To investigate the anti-aging effects of SCP, 20-month-old C57BL/6 J mice were randomly allocated into three groups and subjected to daily intragastric administration for 8 weeks: the aging-control group(physiological saline), a low dose of SCP(500 mg/kg/d)(aging-SCP low), or a high dose of SCP(1000 mg/kg/d)(aging-SCP high). Young mice administered with physiological saline were designated as the young-control group. Following the 8-week intervention period, the anti-aging efficacy of SCP were systematically evaluated through behavioral tests, metabolite analysis, and gut microbiota analysis. Body weight measurements were recorded and organ coefficients were determined for all experimental subjects. The results indicated that, compared with aging-control mice, the brain index of aging-SCP high mice increased (Table 2). Furthermore, biochemical analysis of hepatic function markers AST and ALP demonstrated significantly reduced in both aging-SCP low and aging-SCP high mice compared to aging-control mice (Figure S1). These findings suggest that SCP exhibits therapeutic potential in ameliorating age-related liver function decline. However, SCP showed negligible effects on attenuating the body weight reduction associated with aging (Figure S2). The H&E staining results showed that SCP did not show toxicity in the heart, liver, spleen, lung and kidney of mice, indicating that SCP was safe for mice (Fig. 2).

**Table 2:** The organ coefficients of mouse

Groups(n = 6)	Organ coefficients of aging mice				
	Kidney	Liver	Heart	Lung	Brain
Young-normal	1.10 ± 0.11	4.32 ± 0.63	0.49 ± 0.08ᵃ	0.65 ± 0.10	1.53 ± 0.17ᵃᵇ
Aging-control	1.52 ± 0.23	4.16 ± 1.01	0.47 ± 0.02ᵃ	0.79 ± 0.15	1.40 ± 0.17ᵃ
Aging-SCP low	1.46 ± 0.23	4.44 ± 0.49	0.50 ± 0.05ᵃ	0.84 ± 0.13	1.55 ± 0.12ᵃᵇ
Aging-SCP high	1.57 ± 0.19	4.74 ± 1.39	0.56 ± 0.06ᵇ	1.06 ± 0.72	1.65 ± 0.19ᵇ

^*^Different lowercase letters in the same column indicate significant differences within group (P < 0.05)

### SCP alleviates age- related anxiety in aging mice

**Fig. 3:**
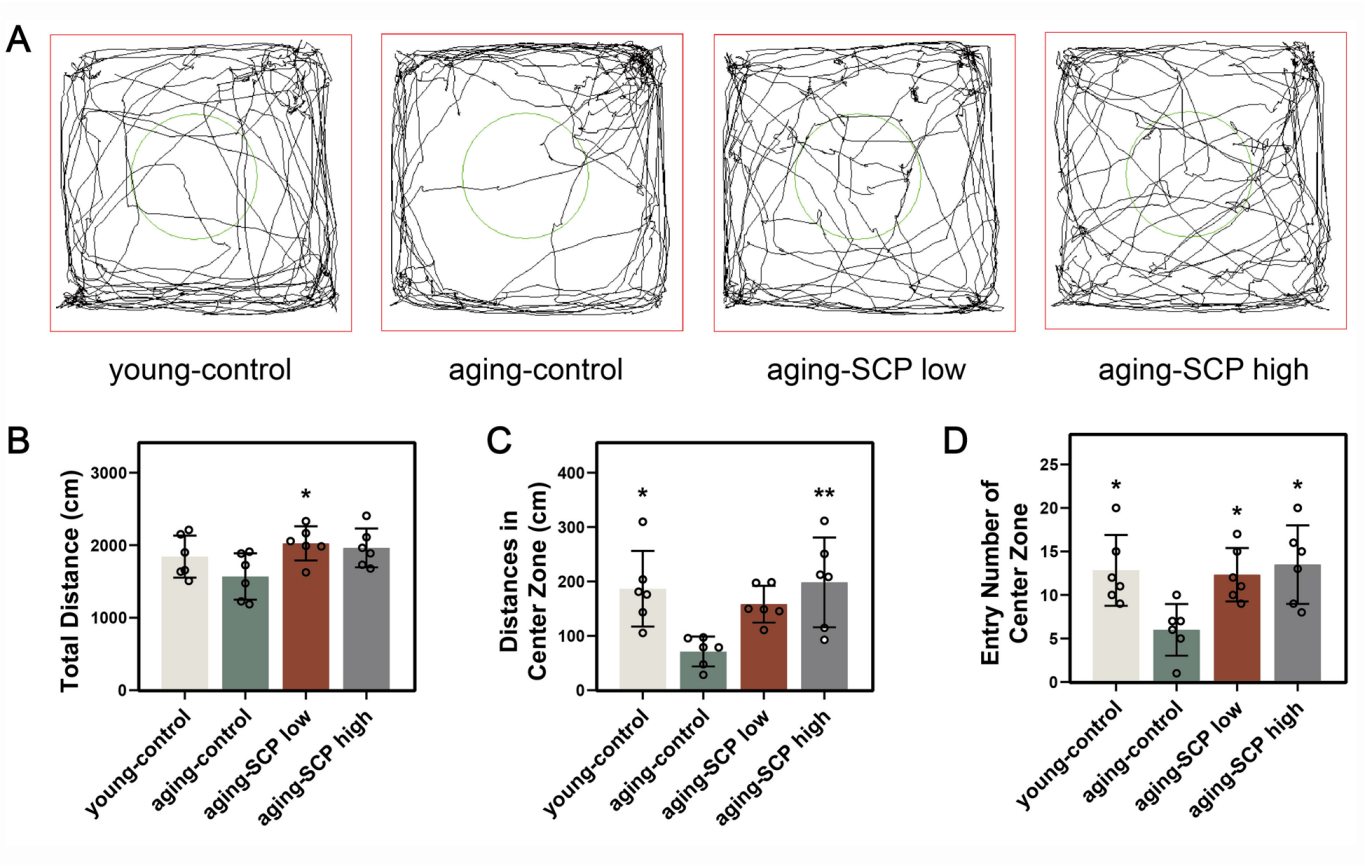
Effect of SCP on autonomic motor ability and anxiety-like behavior of aging mice. **A** representative traces of mice in different groups; **B** total traveled distance; **C** distance in center zone; **D** the number of across the center zone. Comparing with aging-control group. *p < 0.05, **p < 0.01, ***p < 0.001

To evaluate the behavioral impacts of SCP on aging mice, we utilized the open field test to evaluate their spontaneous locomotor activity and exploratory tendencies. The total movement paths, traversal patterns within the central area, and number of entries into the central area were quantitatively recorded for each group of mice. The findings revealed that young mice, aging-SCP low, and aging-SCP high mice displayed longer movement paths and a broader range of movement within the given timeframe, showing a preference for exploring the central area. Conversely, aging-control mice reduced ambulatory distance and tended to stay near the periphery of the open field, indicating that SCP could enhance the spontaneous locomotor activity in aging mice (Fig. 3A). Moreover, the locomotor activity zones of mice can effectively indicate their anxiety levels. Statistical analysis showed that young mice, aging-SCP low, and aging-SCP high mice explored the central area more frequently and covered longer distances within, compared to aging-control mice (Fig. 3B-D). These findings imply that aging may trigger anxiety-like behavior in mice, whereas oral administration of SCP appears to mitigate aging-related anxiety phenotypes.

### SCP attenuates the decline of spatial learning and memory abilities in aging mice

**Fig. 4:**
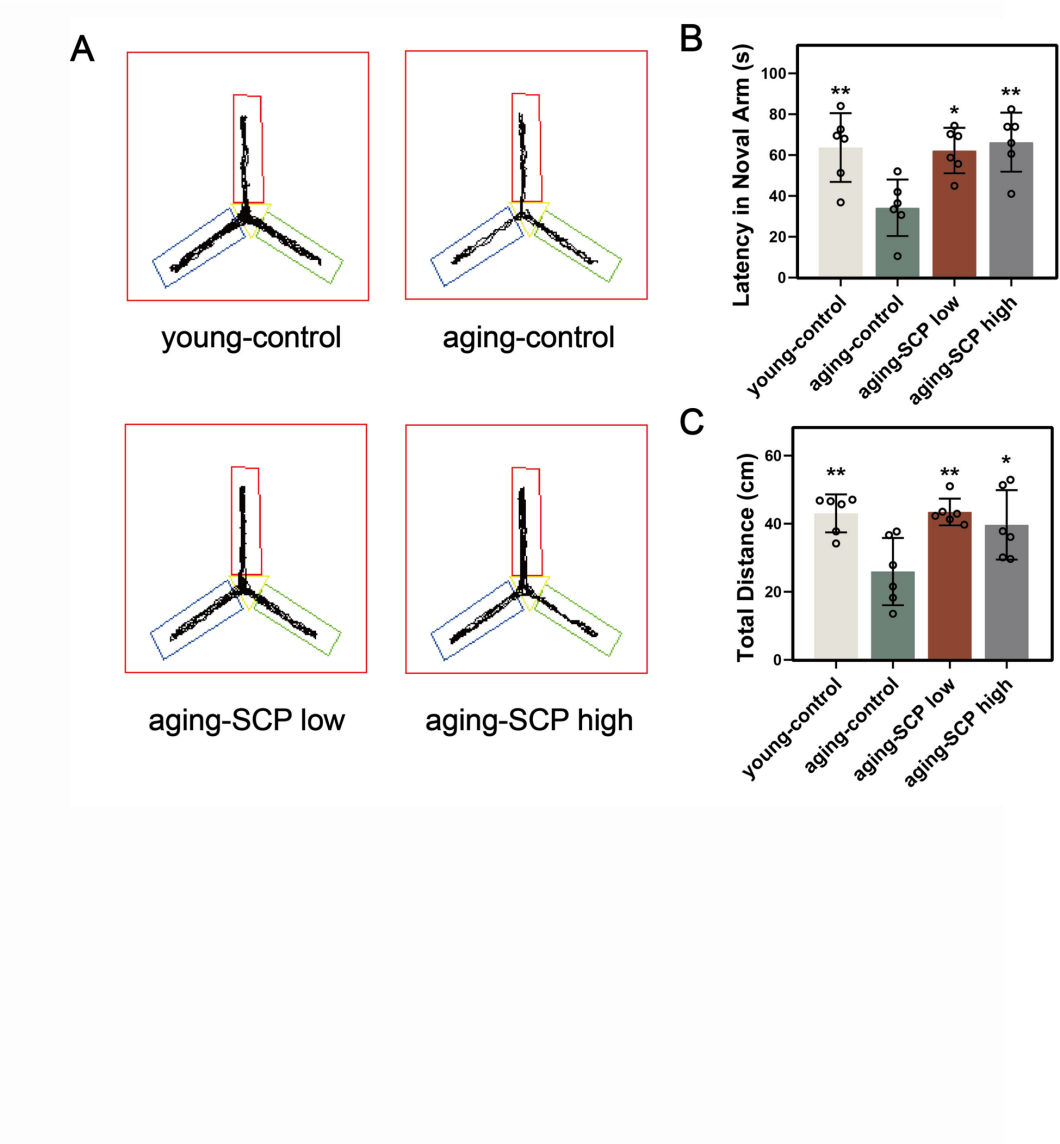
Y maze test evaluates the effect of SCP on working memory of aging mice. **A** representative traces; **B** latency in noval arm; **C** total distance of mice in different groups, when compared with aging-control group. *p < 0.05, **p < 0.01, ***p < 0.001

To further investigate the cognitive influence of SCP in aging mice, the Y-maze test was utilized to evaluate working memory. This behavioral test revealed that aging-control mice significantly reduced total movement distance and time spent in the testing area compared to young-control mice, indicative of a decline in age-associated working memory proficiency. Conversely, experimental analysis revealed that both aging-SCP low and aging-SCP high mice exhibited notably increased movement paths and durations in the testing area relative to aging-control mice, suggesting SCP’s potential in ameliorating age-related working memory deficits (Fig. 4).

**Fig. 5:**
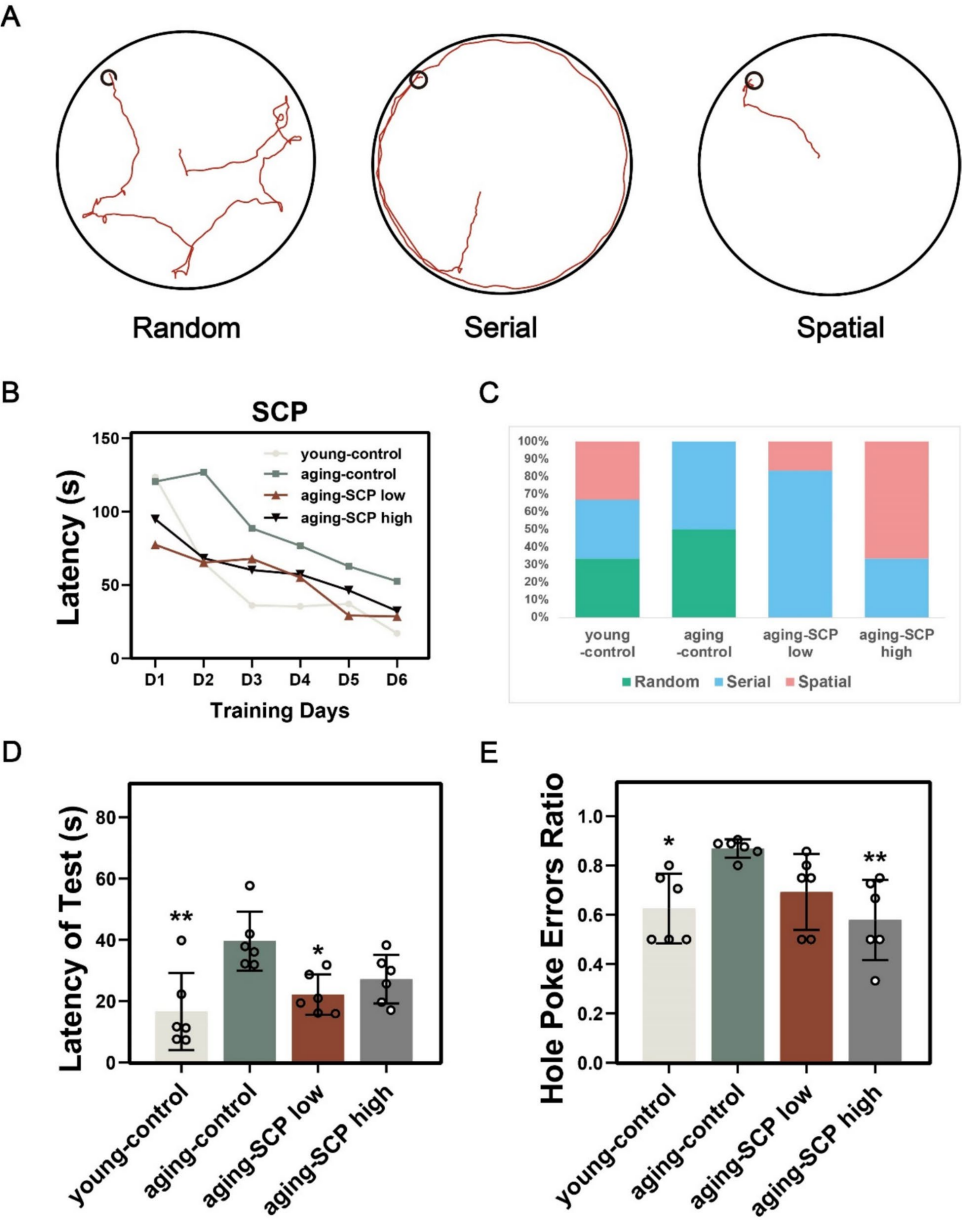
SCP improves cognitive performance of aging mice in the Barnes maze task. **A** examples path plots of random, serial, and spatial trials with state number overlayed on mice tracking data; **B** mean latency of mice in the training phase of the Barnes maze tests (6 consecutive days); **C** aging-control mice predominantly utilized random and serial strategies, while aging-SCP low and aging-SCP high mice favored serial and spatial strategies to find the target hole; **D** and **E** time spent in exploring the target hole(**D**) and the hole poke errors ratio (**E**) of mice in the probe phase of the Barnes maze tests. Comparing with aging-control group. *p < 0.05, **p < 0.01, ***p < 0.001

Moreover, barnes maze outcomes revealed three distinct search strategies: random, serial, and spatial patterns for exploring holes (Fig. 5A). Over the initial six days of training, the time taken by all groups to locate the escape hole gradually decreased (Fig. 5B). Notably, during the testing phase, young mice demonstrated a diverse exploration strategy repertoire, while aging-control mice predominantly utilized random and serial strategies. In contrast, aging-SCP low and aging-SCP high mice favored serial and spatial strategies, with approximately 70% of aging-SCP high mice opting for the most efficient spatial strategy (Fig. 5C). Statistical analysis demonstrated that young-control, aging-SCP low, and aging-SCP high mice exhibited reduced exploration times and fewer errors compared to aging-control mice (Fig. 5D and E), indicative of superior spatial learning and memory abilities. These results demonstrate that SCP administration significantly attenuates age-related cognitive decline in learning and memory functions, supporting its potential as a cognitive-enhancing intervention for aging populations.

### Protective efficacy of SCP on the brain tissue of aging mice

**Fig. 6:**
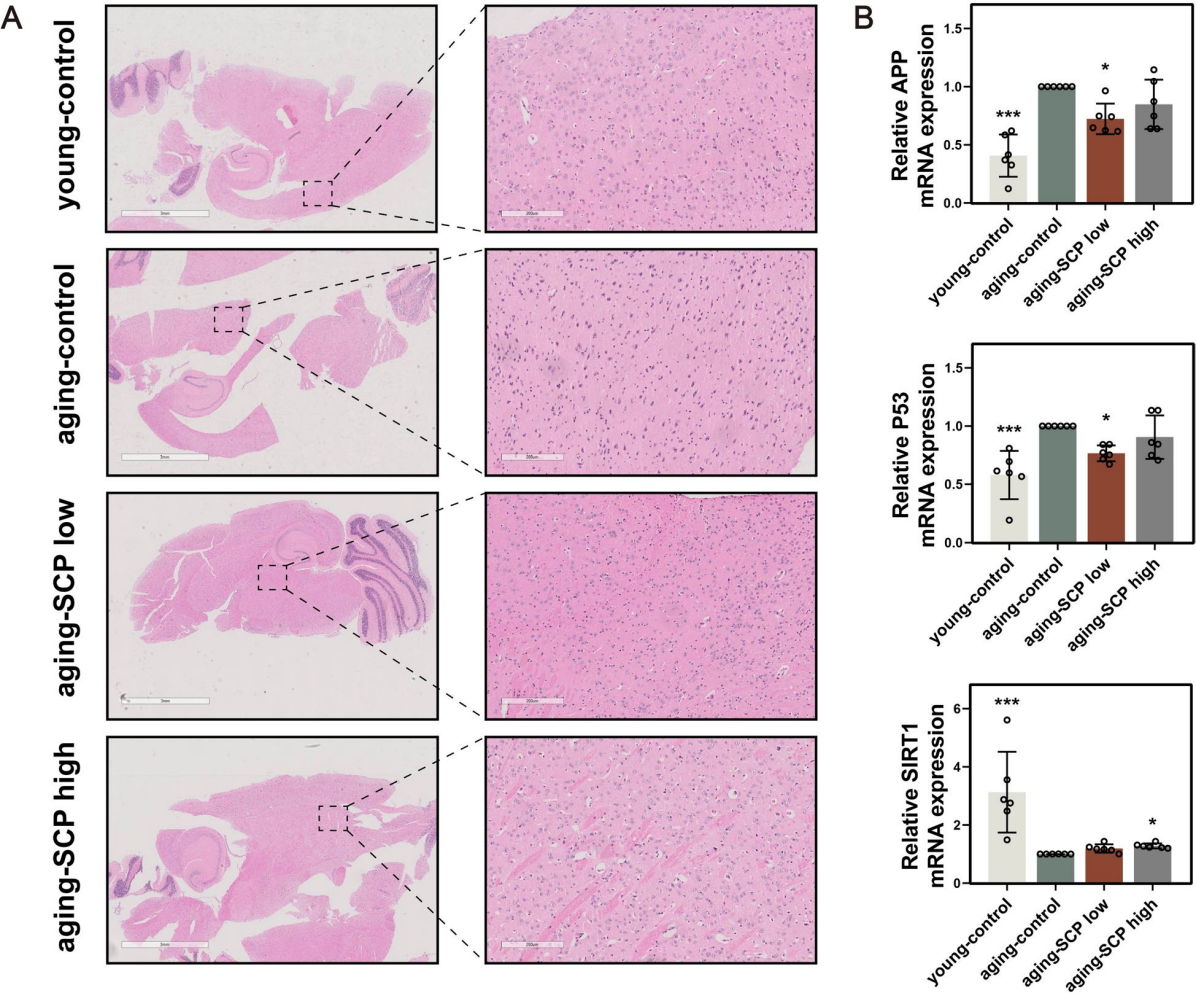
SCP could enhance neuronal cell numbers and suppress the expression of aging-related genes. **A** Representative picture of H&E staining in different mice brain tissues; **B** mRNA expression of the aging-associated genes APP, P53 and the anti-aging gene SIRT1 in different mice brain tissues. Comparing with aging-control group. *p < 0.05, **p < 0.01, ***p < 0.001. Scale bar: 3 mm (left) and 200 μm (right)

Hematoxylin and eosin(H&E) staining was utilized to systematically evaluate any pathological alterations in the brain tissue across four groups of mice. Compared to the young-control mice, a noticeable reduction in neuronal density was observed in aging-control mice. SCP treatment preserved neuronal density, with aging-SCP low and aging-SCP high mice exhibited a significantly higher number of neuronal cells than the aging-control group, indicating SCP's potential to attenuate age-associated neuronal loss (Fig. 6A). Furthermore, RT-qPCR analysis demonstrated significant upregulation of aging-associated genes APP and P53 in all other three groups as opposed to the aging-control mice, alongside reduced expression of the anti-aging gene SIRT1(Fig. 6B). These findings suggest that SCP could ameliorate age-related cerebral dysfunction by enhancing neuronal cell numbers and suppressing the expression of aging-related genes.

### SCP optimizes gut microbiota composition in aging mice

**Fig. 7:**
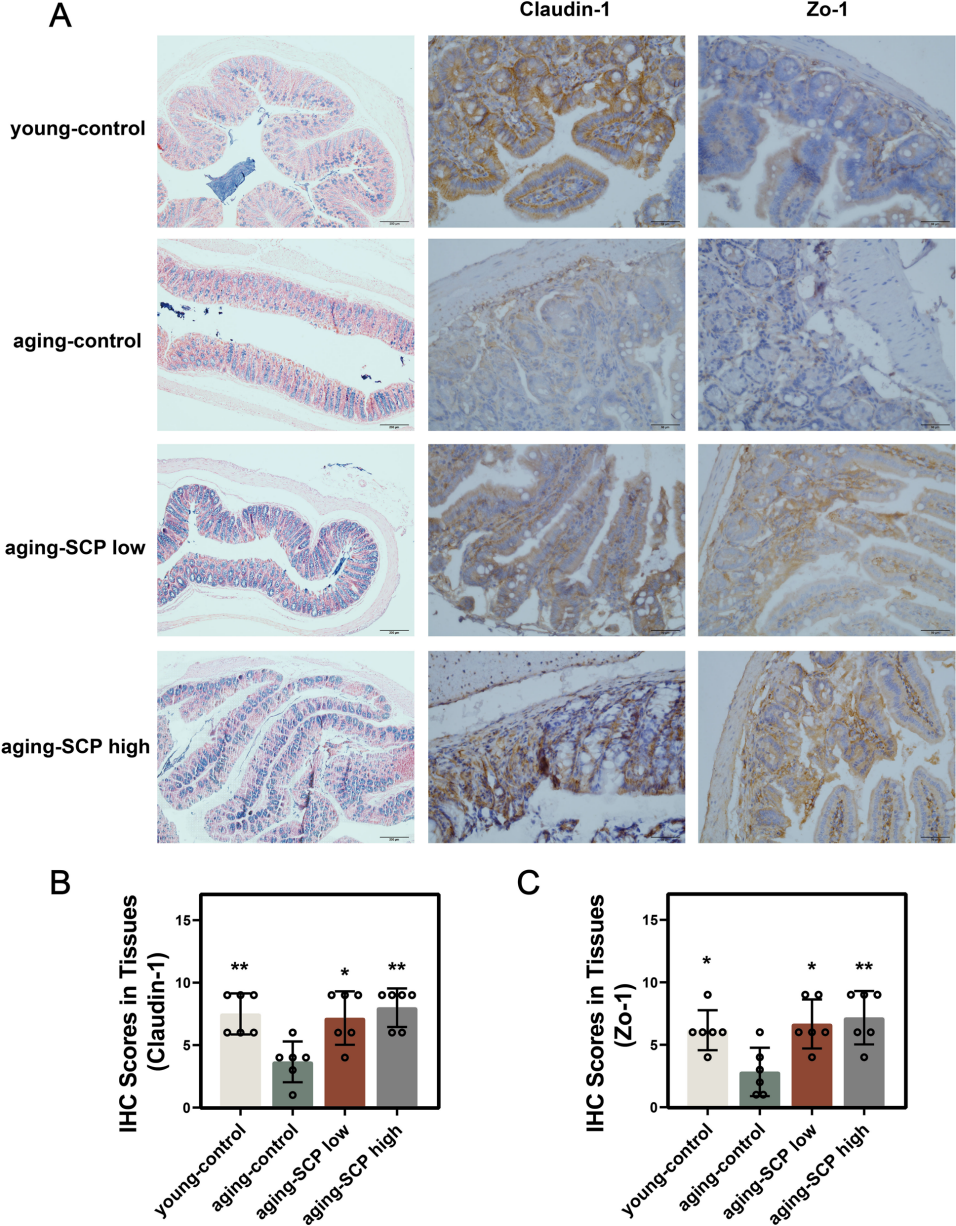
The intestinal function has improved. **A** Representative AB and IHC images of the different mice groups; **B** and **C** The scores of the representative proteins Claudin-1 (**B**) and Zo-1 (**C**) of tight junction proteins. *p < 0.05, **p < 0.01, ***p < 0.001. Scale bar: 200 μm (left) and 50 μm (middle and right)

Aging, a progressive physiological deterioration in the body, has been strongly linked to alterations in gut microbiota [10]. In this study, AB staining and immunohistochemical staining of intestinal tissues were performed. The results revealed that, compared with the aging-control group, mice fed with SCP exhibited a significant increase in the number of goblet cells and enhanced secretion of acidic mucins in the intestinal tissue, suggesting that SCP may improve intestinal mucosal barrier function in aging mice (Fig. 7A). The intestinal mechanical barrier is composed of the basement membrane, epithelial cells, and the overlying mucus layer [11]. Among these components, epithelial cells maintain barrier integrity primarily through tight junctions. Zo-1 and Claudin-1 play crucial roles in the composition of tight junction proteins. Our study demonstrated that, compared with the aging-control group, both the aging-SCP low and aging-SCP high groups exhibited significantly increased protein expression levels of Zo-1 and Claudin-1 in intestinal tissues. These findings indicate that SCP supplementation effectively enhances intestinal barrier function in aging mice (Fig. 7A–C). We then systematically examined SCP's modulatory effects on the aging mice gut microbiome through high-throughput sequencing of the 16S rRNA gene V3-V4 hypervariable regions via Illumina NovaSeq for bacterial community structure analysis. Through species annotation, we identified and compared species at the phylum level across all groups and generated stacked bar charts depicting relative species abundances.

**Fig. 8:**
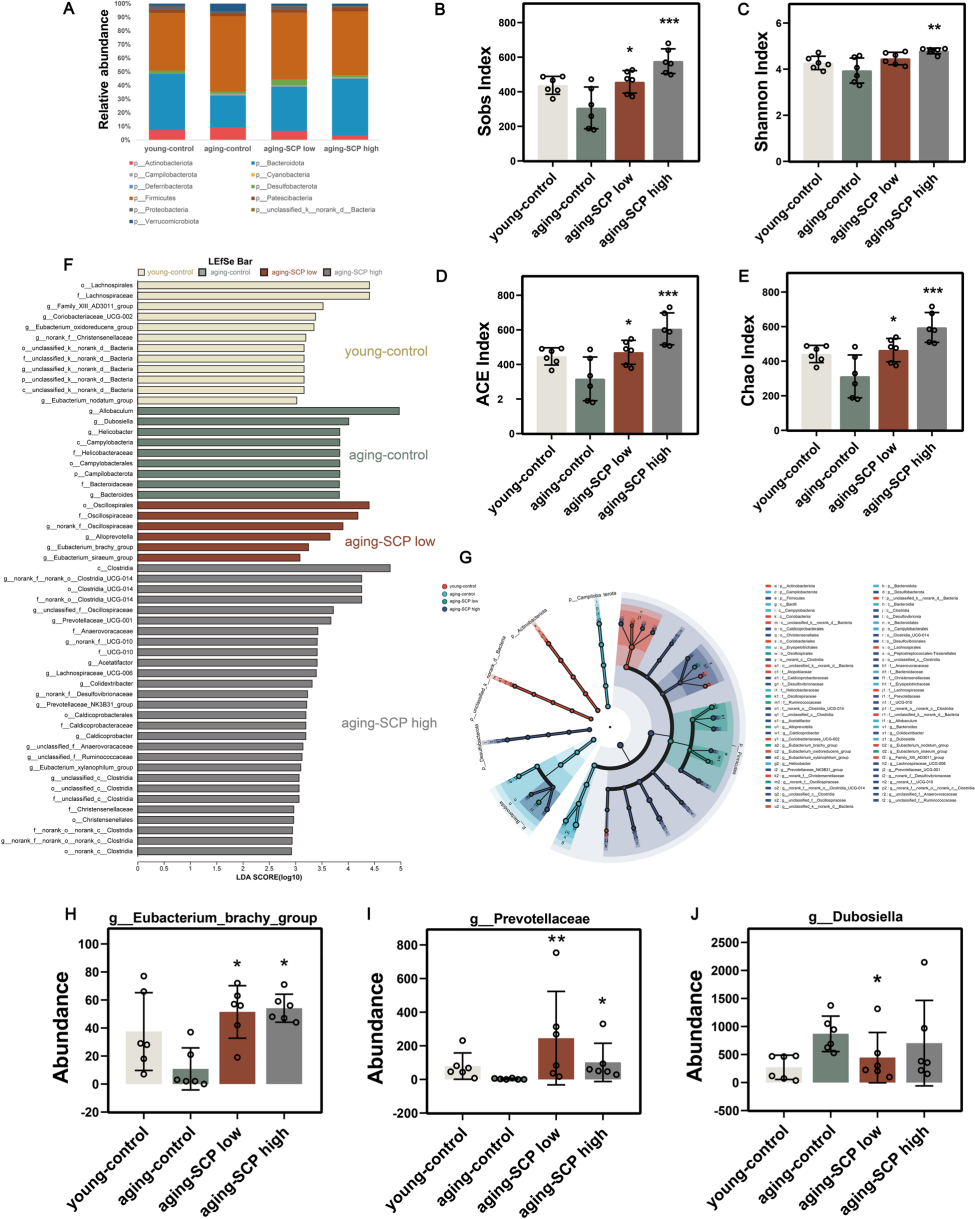
SCP alters the gut microbiota structure in aging mice. **A** Relative abundance of phylum columnar cumulative plot. **B**-**E** The diversity indices (Sobs, Shannon, ACE and Chao) of different mice; **F** LEfSe, LDA Effect Size. **G** LEfSe, branching diagram; **H**-**I** The abundance of g_Eubacterium_brachy_group (**H**), g_Prevotellaceae (**I**) and g__Dubosiella (**J**) at the genus level. Comparing with aging-control group. *p < 0.05, **p < 0.01, ***p < 0.001

The results revealed notable differences in species composition at the phylum level among the experimental groups. Specifically, the aging-control group exhibited significant Firmicutes/Bacteroidetes(F/B) ratio dysregulation, characterized by a marked increase in Firmicutes abundance and a concurrent decrease in Bacteroidetes compared to the young-control group. Remarkably, SCP intervention significantly normalized the Firmicutes to Bacteroidetes ratio, aligning the gut microbiota structure toward youthful levels (Fig. 8A). The Firmicutes to Bacteroidetes ratio is a validated biomarker of the gut microbiota's equilibrium, suggesting that SCP possesses the capacity to counteract age-associated dysbiosis.

Furthermore, key diversity and richness indices including Shannon, Chao1, ACE, and Sobs exhibited a substantial increase in the gut microbiota of aging mice following SCP administration, as illustrated in Fig. 8B–E. To identify group-specific microbial biomarkers, we performed a Lefse analysis(LDA Score = 4). Differential abundance analysis identified significant taxonomic shifts in aging-SCP low and aging-SCP high groups in contrast to the aging-control group, notably marked by an upsurge in Clostridiales in the High SCP group and an increase in Oscillospirales in the Low SCP group (Fig. 8F and G). At the genus level, enrichment of g_Eubacterium_brachy_group and g_Prevotellaceae was evident in both the aging-SCP low and aging-SCP high groups, whereas g__Dubosiella did not exhibit a noticeable trend towards enrichment (Fig. 8H–J). Notably, literatures suggest that Eubacterium, Prevotellaceae, and Dubosiella are closely linked to the development and progression of neurodegenerative diseases such as Alzheimer's and Parkinson's disease [12–14]. Integrative analysis imply that SCP can restores age-disrupted gut microbial ecology, indicating gut-brain axis mediation of its geroprotective effects.

### SCP ameliorates the metabolic disorders of aging mice

**Fig. 9:**
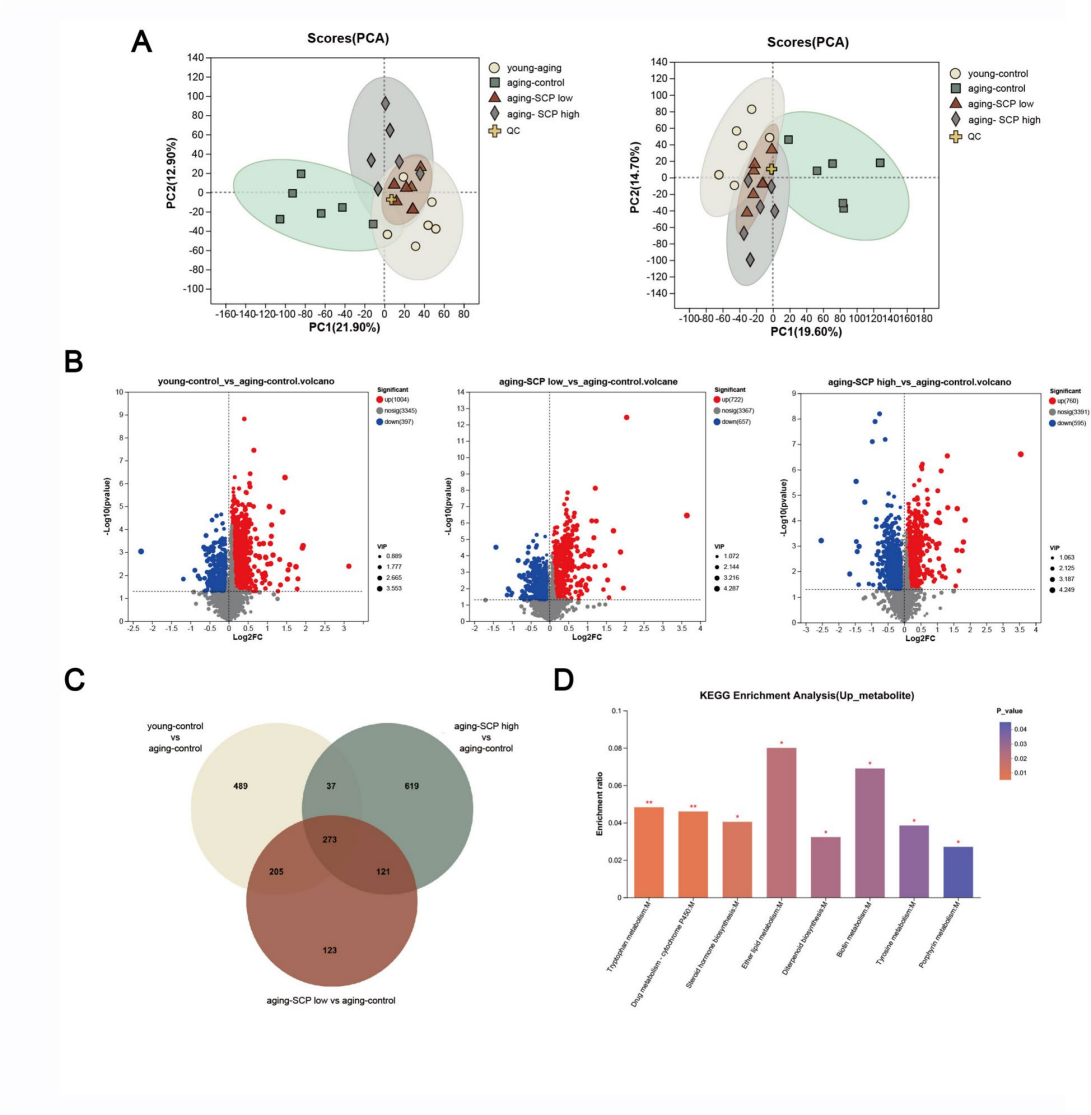
SCP regulates metabolic disorders in aging mice. **A** PCA, Principal Component Analysis; **B** Volcano plot; **C** 273 metabolites were upregulated in the aging-SCP low group, aging-SCP high group, and young-control group when compared with aging-control group. **D** Kyoto Encyclopedia of Genes and Genomes (KEGG) path annotation of differential metabolites. *p < 0.05, **p < 0.01

To systematically evaluate SCP's modulatory effects on age-related metabolic alterations, we conducted comprehensive fecal metabolomic profiling using metabolomics techniques. Fecal samples were collected from all mice prior to the conclusion of the experiment and subjected to LC–MS/MS analysis for metabolomics investigation. In order to assess the impact of SCP on aging mice, Principal Component Analysis(PCA) was employed to evaluate the metabolite expression profiles across the four experimental groups. The PCA results revealed a significant metabolic disparity between the young-control and aging-control groups in both positive and negative ion modes, indicating substantial differences in aging-related metabolite expression profiles. However, post-SCP intervention, a closer alignment in metabolite expression profiles was observed among the aging-SCP low, aging-SCP high, and young-control groups (Fig. 9A), suggesting SCP's potential in normalizing age-disrupted metabolic profiles. This finding was further supported by the Partial Least Squares Discriminant Analysis(PLS-DA) outcomes(Figure S3).

**Fig. 10:**
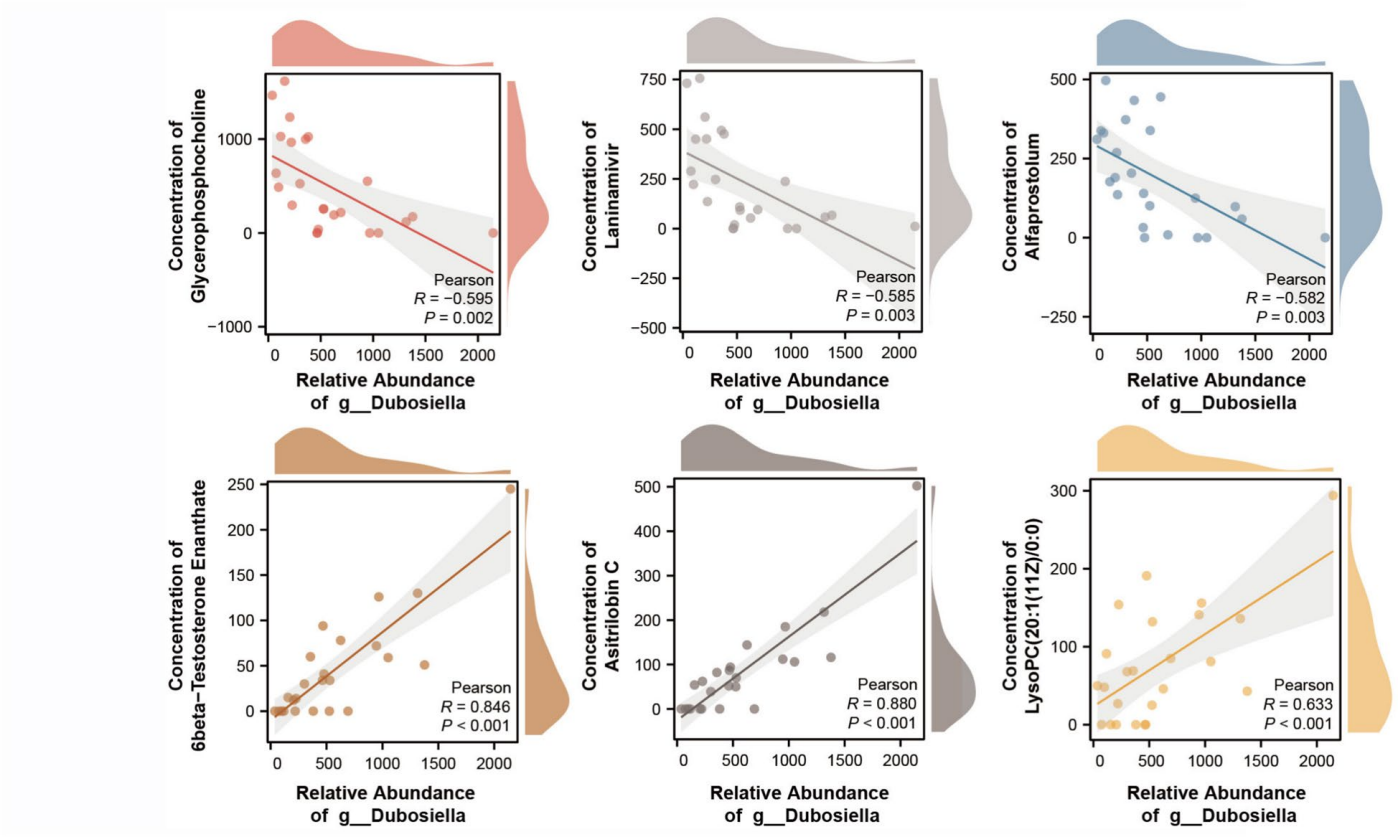
The correlation analysis between the abundance of gut microbiota(g__Dubosiella) and the concentration of host metabolites(Glycerophosphocholine, Laninamivir, Alfaprostolum, 6 β-Testosterone enanthate, Asitrilobin C, and LysoPC(20:1(11Z)/0:0)) after the SCP intervention

Differential analysis revealed significant variances in the metabolite expression profiles of the aging-SCP low group, aging-SCP high group, and young-control group compared to the aging-control group. A comprehensive screening for differential metabolites in these groups relative to the aging-control group was conducted (Fig. 9B). Integrative analysis identified 273 metabolites that were significantly upregulated in both SCP-treated groups and young-control group relative to age-control group (Fig. 9C). Through KEGG pathway annotation, these metabolites were notably enriched in the steroid hormone biosynthesis pathway (Fig. 9D). Furthermore, a correlation analysis was performed between gut microbiota and host metabolites following SCP intervention. The findings revealed a negative correlation of Dubosiella with Glycerophosphocholine, Laninamivir, and Alfaprostolum, among others, while showing a positive correlation with 6 β-Testosterone enanthate, Asitrilobin C, LysoPC(20:1(11Z)/0:0), and others (Fig. 10). These results highlight a strong correlation between the alteration in Dubosiella abundance within the intestine and the enhancement of metabolic patterns post-SCP intervention.

**Fig. 11:**
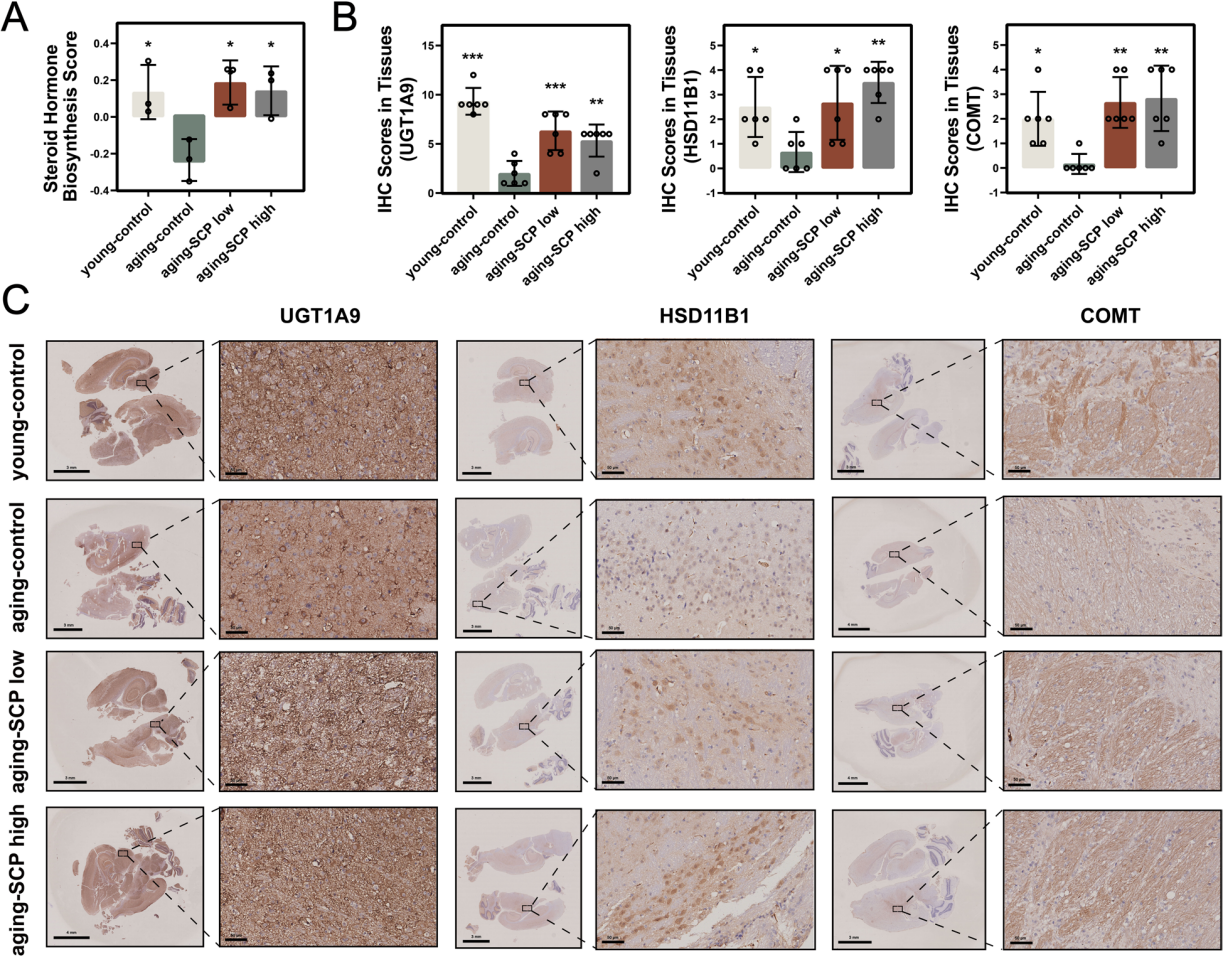
Steroid hormone biosynthesis pathway was highly expressed in brain tissue. **A** the GSVA score of steroid hormone biosynthesis pathway; **B** The IHC scores of the representative proteins COMT, UGT1A9 and HSD11B1 of steroid hormone biosynthesis pathway; **C** Representative IHC images of the different mice groups. *p < 0.05, **p < 0.01, ***p < 0.001. Scale bar: 3 mm (overall images) and 50 μm (enlarge images)

To further verify that SCP can activate the steroid hormone biosynthesis pathway, we employed RNA-seq to detected the gene expression profiles of brain tissues. GSVA analysis was used to calculate the steroid hormone biosynthesis score according to the genes set associated with this metabolic pathway. The results showed that the steroid hormone biosynthesis score appeared a low trend in the aging-control group, which showed the opposite trend in the young-control, aging-SCP low and aging-SCP high group (Fig. 11A). IHC analysis revealed significant upregulation of key proteins involved in steroid hormone biosynthesis COMT, UGT1A9 and HSD11B1 in both aging-SCP low and aging-SCP high group compared to aging-control group (Fig. 11B–C). Taken together, these observations suggest that SCP may delay brain tissue decline in aging mice by promoting steroid hormone biosynthesis.

## Discussion

Aging is the consequence of complex interactions between genetic and environmental factors [15]. Accumulating pharmacological evidence establishes the pivotal role of the gut microbiota as an intrinsic regulatory element in human aging [10, 16]. The human gut microbiome, described as a symbiotic"superorganism"of co-evolved microorganisms with the human body, represents a second genome beyond the human genome and significantly contributes to maintaining human health [17]. Through intricate interactions with the body, the gut microbiota orchestrates a unique microenvironment within the human system, actively participating in disease regulation [18, 19]. Notably, the composition and function of the microbiota are substantially influenced by dietary regimens [20]. As such, exploring pharmaco-nutritional interventions capable of rebalancing the gut microbiota represents a strategically significant approach with substantial translational potential in delaying the aging process.

Sea cucumbers are pharmacologically prized resources, containing a plethora of bioactive constituents that have captured significant attention for their potential in nutraceutical formulations and pharmaceutical development. Among these constituents, sea cucumber peptides demonstrate as effective bioactive agents. A previous comprehensive assessment of the biological properties of sea cucumber peptides revealed their antioxidant, antihypertensive, antihyperuricemic, antitumor, alleviating gut microbiota imbalance, antiaging, antifatigue, hypoglycemic, collagen-boosting, anti-inflammatory, and immunomodulatory properties [9]. This study advanced beyond prior sea cucumber research—primarily focused on extraction optimization and isolated bioactivities—by first elucidating SCP's gut-brain axis-mediated neuroprotective mechanisms in aging. We further established a novel multi-omics framework (microbiome-metabolome-transcriptome-phenotype) to mechanistically dissect SCP's geroprotective effects, providing a paradigm for modernizing traditional nutraceuticals.

In this investigation, a naturally aging C57BL/6 J mouse model was employed to explore the potential of SCP in modulating age-related physiological decline. Our study demonstrated that oral administration of SCP enhanced liver function-related parameters AST and ALP levels, indicating the potential of SCP in ameliorating aging-induced liver function decline in geriatric populations. The results of the open field test illustrated that the mice, following oral SCP administration, exhibited increased movement paths, wider motion range, and higher propensity to explore the central area, indicative of SCP's potential in ameliorating age-related declines in motor skills and anxiety symptoms. Analysis from the Y-maze test demonstrated that SCP-intervened mice displayed notable enhancements in movement paths and durations within the testing area compared to aging-control counterparts, suggesting SCP's efficacy in mitigating age-related declines in working memory. Similarly, findings from the Barnes maze test indicated that SCP effectively attenuated the cognitive deficits in learning and memory functions induced by aging in mice. Moreover, complementary analyses from H&E staining and RT-qPCR demonstrated that oral SCP administration elevated the neuronal count in brain tissue and significantly downregulated aging-related gene expression profiles. Collectively, these findings underscore SCP's capacity to address aging-induced impairments in motor skills, cognitive function, memory, and anxiety, alongside its potential to ameliorate declines in brain tissue functionality.

The bidirectional communication between gut microbiota and cerebral functions has been extensively documented in the literature [21]. Previous research has demonstrated the ability of Dimethyl itaconate to modulate intestinal microbial balance, thereby ameliorating cognitive impairment in mice affected by a high-fat diet via the gut-brain axis [22]. Our investigation revealed that SCP intervention significantly enhanced microbial diversity and richness in aged mice subjects. Of particular significance, SCP administration effectively restored the age-associated dysbiosis of the Firmicutes/Bacteroidetes ratio—a recognized biomarker of gut ecosystem imbalance. This phylum-level reorganization holds clinical relevance given established correlations between Firmicutes abundance variations and neurodegenerative pathologies including Parkinson’s (PD) and Alzheimer's diseases (AD) [23]. Substantiating this connection, Hou et al*.* documented Firmicutes elevation in PD models that was subsequently normalized following osteocalcin treatment [24]. Mechanistically, our results demonstrate that SCP-mediated Firmicutes suppression in senescent mice underscores its geroprotective potential through microbial modulation. Taxonomical analysis identified three key bacterial genera—Eubacterium_brachy_group, Prevotellaceae, and Dubosiella—as principal mediators of SCP's anti-aging effects. Existing literature notes the dysregulation of Eubacterium and Dubosiella in Alzheimer's disease models [12], as well as the downregulation of Prevotellaceae in Parkinson's disease patients [25]. Prevotellaceae members function as crucial gut symbionts, and their reduction is linked to increased exposure to bacterial endotoxins and escalated intestinal permeability, ultimately leading to uncontrolled expression and misfolding of colonic α-Synuclein [26]. Our findings propose that targeted modulation of these microbial populations through SCP supplementation may represent a novel non-pharmacological strategy for mitigating age-related neurological decline and metabolic dysregulation. Supporting this therapeutic potential, metabolomic profiling revealed that SCP intervention shifted aged subjects'metabolic signatures toward younger phenotypic patterns. This multimodal evidence positions SCP as a promising nutraceutical candidate for preserving gut-brain axis integrity and counteracting age-associated pathophysiological processes.

In comparison to the aging-control group, metabolites notably upregulated in the aging-SCP low and aging-SCP high groups exhibit enrichment in the steroid hormone biosynthesis pathway. Steroids play a vital role in the nervous system, boosting neuronal vitality, contributing to myelin formation, and impacting cognitive functions, particularly learning and memory capabilities [27]. Validation through RNA-seq and IHC staining results confirmed that the steroid hormone biosynthesis score and the expression of three key genes included in steroid hormone biosynthesis pathway were elevated in the aging-SCP low and aging-SCP high groups. This evidence suggests that SCP promotes steroid synthesis to mitigate the decline in brain tissue function in aging mice.

In conclusion, our investigation employing a physiologically aged mice model with multi-omics integration elucidates the geroprotective mechanisms of SCP through gut-brain axis modulation. Critically, SCP administration mitigated age-related dysbiosis in both microbial communities and metabolic profiles, particularly through restoration of neuroprotective microbial taxa and steroidal metabolite biosynthesis. These collective findings provide mechanistic insights into SCP's capacity to counteract cerebral aging by modulating gut-brain axis homeostasis, while establishing translational frameworks for developing novel therapeutic interventions against age-related neurological disorders. Further mechanistic studies encompassing targeted metabolite quantification and microbiota transplantation are warranted to validate the causal relationships identified in this pioneering work.

## Conclusions

Our findings establish that SCP may function as a geroprotective compound through precision modulation of the gut-brain axis in physiologically aged mice models, providing mechanistic insights into its translational potential for enhancing healthy longevity in humans.

## Supplementary Information

Additional file 1

## Data Availability

The data used to support the findings of this study are available from the corresponding author upon request.

## Abbreviations

SCP: Sea cucumber polypeptide
HPLC: High performance liquid chromatography
UV: Ultraviolet
TSK: Tosoh silica
UPLC-Q-TOF–MS: Ultra-performance liquid chromatography coupled with quadrupole time of flight mass spectrometry
LC–MS/MS: Liquid chromatography tandem mass spectrometry
UHPLC: Ultra high performance liquid chromatography
KEGG: Kyoto Encyclopedia of Genes and Genomes
DNA: Deoxyribonucleic acid
PCR: Polymerase chain reaction
RT-qPCR: Real-time reverse transcription quantitative polymerase chain reaction
mRNA: Messenger ribonucleic acid
AST: Aspartate aminotransferase
ALP: Alkaline phosphatase
H&E: Hematoxylin and eosin
rRNA: Ribosomal ribonucleic acid
PCA: Principal component analysis
PLS-DA: Partial least squares discriminant analysis
