# Supplementary material for: Sea cucumber polypeptide ameliorates aging properties via the brain-gut axis in naturally aging mice
Source: Chin Med. 2025 Aug 29;20:136. doi: 10.1186/s13020-025-01201-2 (PMC12395763; doi:10.1186/s13020-025-01201-2)
Supplement: Supplementary file 1 — Additional file 1 [file 13020_2025_1201_MOESM1_ESM.docx]

**Table S1.** The distribution of different molecular weight SCP

| Molecular Weight | Content (%) |
| --- | --- |
| < 2000 | 98.72 |
| < 1000 | 92.88 |
| < 500 | 73.47 |

**Table S2.** The amino acid composition of SCP

| Amino Acid Types | Content(g/100g) | Amino Acid Types | Content(g/100g) |
| --- | --- | --- | --- |
| Asp | 6.49 | Met | 0.64 |
| Thr | 2.64 | Ile | 1.72 |
| Ser | 2.24 | Leu | 2.50 |
| Glu | 9.26 | Tyr | 1.30 |
| Pro | 6.71 | Phe | 1.05 |
| Gly | 17.1 | Lys | 1.51 |
| Ala | 8.44 | His | 0.38 |
| Val | 2.84 | Arg | 5.34 |

**Table S3.** Sequences of primers used in this study

| **Gene name** | **Sequence (5’-3’)** |
| --- | --- |
| APP | Forward: ACCCACTGATGGTAATGCTG |
|  | Reverse: CACTGGTTGGTTGGCTTCTA |
| P53 | Forward: TGCATGGACGATCTGTTGCTGCC |
|  | Reverse: CAGGTGGAAGCCATAGTTGCCCT |
| SIRT1 | Forward: AACAGGTTGCGGGAATCCAAAGG |
|  | Reverse: CTCCTCGTACAGCTTCACAGTCAAC |
| β-actin | Forward: TTCAACACCCCAGCCATG |
|  | Reverse: CCTCGTAGATGGGCACAGT |


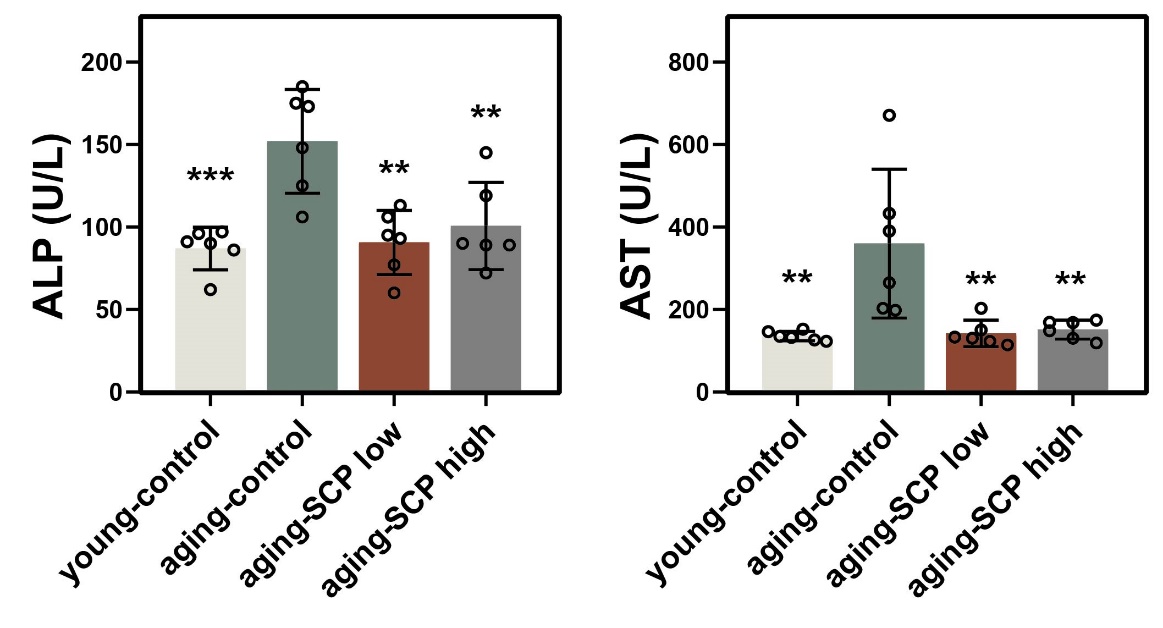


**Figure S1.** Serum levels of aspartate aminotransferase (AST) and alkaline phosphatase (ALP) in different mice. *p <0.05, **p <0.01, ***p <0.001.


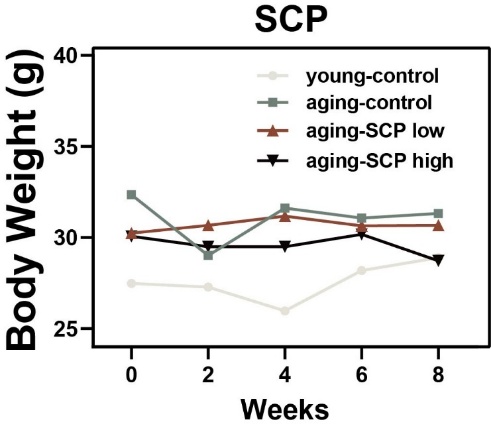


**Figure S2.** The body weight of mice.


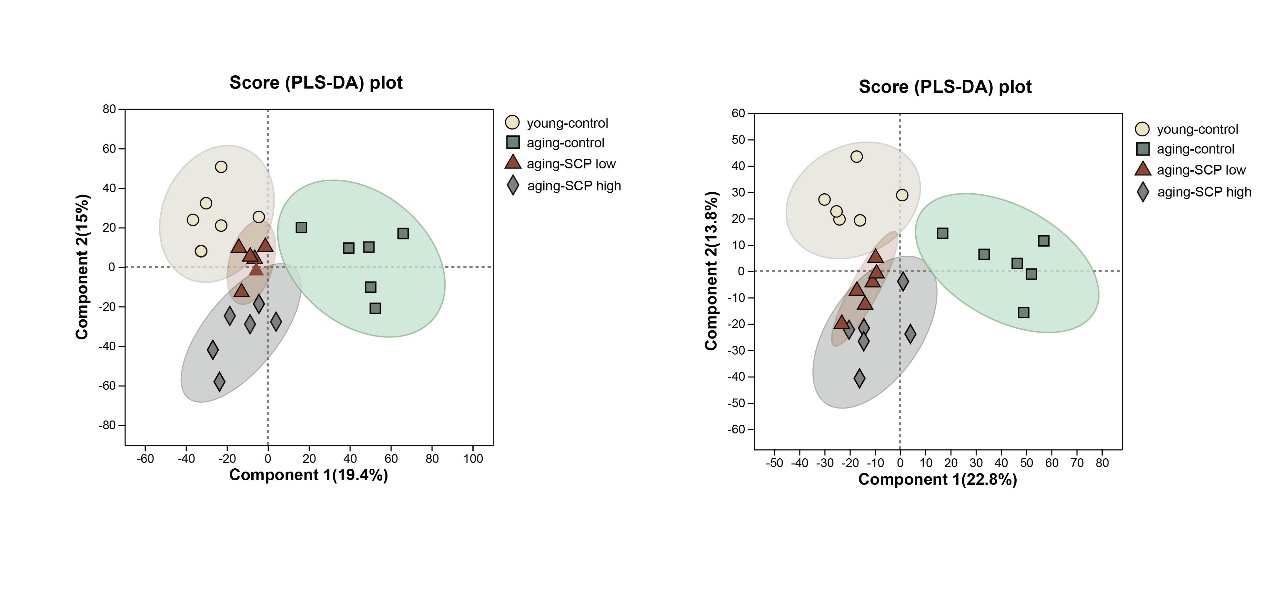


**Figure S3.** The Partial Least Squares Discriminant Analysis (PLS-DA) reveals that SCP can mitigate aging-related metabolic disorders. *p <0.05, **p <0.01, ***p <0.001.
